# Geographical and environmental patterns of Carpathian land snail faunas in a region of high endemicity

**DOI:** 10.1038/s41598-024-51870-6

**Published:** 2024-01-16

**Authors:** Voichița Gheoca, Ana Maria Benedek, Robert Cameron

**Affiliations:** 1 Faculty of Sciences, Applied Ecology Research Center, Lucian Blaga University of Sibiu, 5-7 Raţiu Street, 550012 Sibiu, Romania; 2 Natural History Museum, London, SW7 5BD UK

**Keywords:** Zoology, Environmental sciences

## Abstract

The land snail faunas of limestone gorges of Romanian Carpathians were sampled to test the effect of geographic and environmental factors on the malacofauna richness and composition. A total of 134 sites within 28 limestone gorges were surveyed during 2011–2019 using a combined strategy of visual search and litter/topsoil analysis. Environmental variables such as geographic location, altitude, climate, microhabitat type, dominant vegetation, tree cover and width of the gorge were recorded to detect the relationship with species richness and composition. While the numbers of species, their identities and their abundance varied greatly among samples, both presence and absence data and quantitative multivariate analyses showed that region and climate or altitude (both strongly associated with region) accounted for far more variation than differences in tree cover and dominant microhabitat. Nevertheless, the effects of different habitat preferences were evident. The mixture of species with very restricted ranges within this Pleistocene refugium and those that have spread widely during the Holocene raise questions about the meaning of region when related to local richness and composition.

## Introduction

The Carpathian Mountains, running from Slovakia and Poland in the far west through Ukraine and Romania to a small part of Serbia, have a number of features that distinguish them from the other major mountain ranges in Europe^1^. In particular, their position and range of altitude resulted in a terrain that provided many refugia of a number of habitat types during the recurrent glacial periods of the Pleistocene. Even at maximum extent, glaciers covered less than 1% of the area (details cited in^1^). Woodland persisted in at least the southern parts^2–5^, while alpine habitats, although while fragmented, persisted through warmer interglacials^6^.

The pattern of repeated isolation and expansion of several habitats has given rise to a number of endemic species of both plants and animals; the proportions of such endemics and the extent of their ranges vary both by taxon and with the habitats occupied^1^. At the same time, the Carpathians have been the source of species moving north and west in the Holocene, so many species are more widely distributed in the suitable habitats.

The Carpathian land snail fauna is in many ways typical of this mixture of the wide-ranging and the endemic^7,8^. In a previous study of Transylvanian forest snails^9^, the faunas showed a predominance of widespread species found further north^10^, although there was a slow decay in similarity from northwest to southeast. Forests from the Banat, at the south-western end of the chain, were more distinctive^11,12^; although holding a similar proportion of species widespread in northern and central Europe, they held a higher proportion of species also found further south. While geographical position accounted for some differences between sites, there are many environmental variables involved. In particular, climate, including the effects of aspect, vegetation cover, especially tree cover, and the availability of exposed limestone rock are known to influence the richness and composition of local snail faunas.

Local land snail faunas contain a mixture of those constrained by historical events and poor powers of dispersal and those for which present habitat is the main determinant of their presence and abundance. Their patterns of richness and composition relate to the more general issue of the relationship between local and regional richness^13–17^.

The results of our previous studies conducted in the Romanian Carpathians revealed some interesting differences between the snail fauna of different regions^9,10^, results we decided to explore further at a larger scale. In this study, we have broadened the range of habitats studied to explore the interaction between these factors. While rock type (limestone) is held constant, the altitude, aspect and vegetation cover vary greatly among samples. Samples from a single gorge in Dobrogea, away from the Carpathian chain, serve to provide a contrast to the main series of sites, being isolated from the rest. Our objectives were to: (1) record the characteristics of land snail fauna inhabiting the Charpathian limestone gorges; (2) identify the regional particularities of the limestone malacofauna; (3) explore the contribution of geographic position and environmental variables in shaping the snail fauna.

## Material and methods

### Sampling and recording

**Figure 1:**
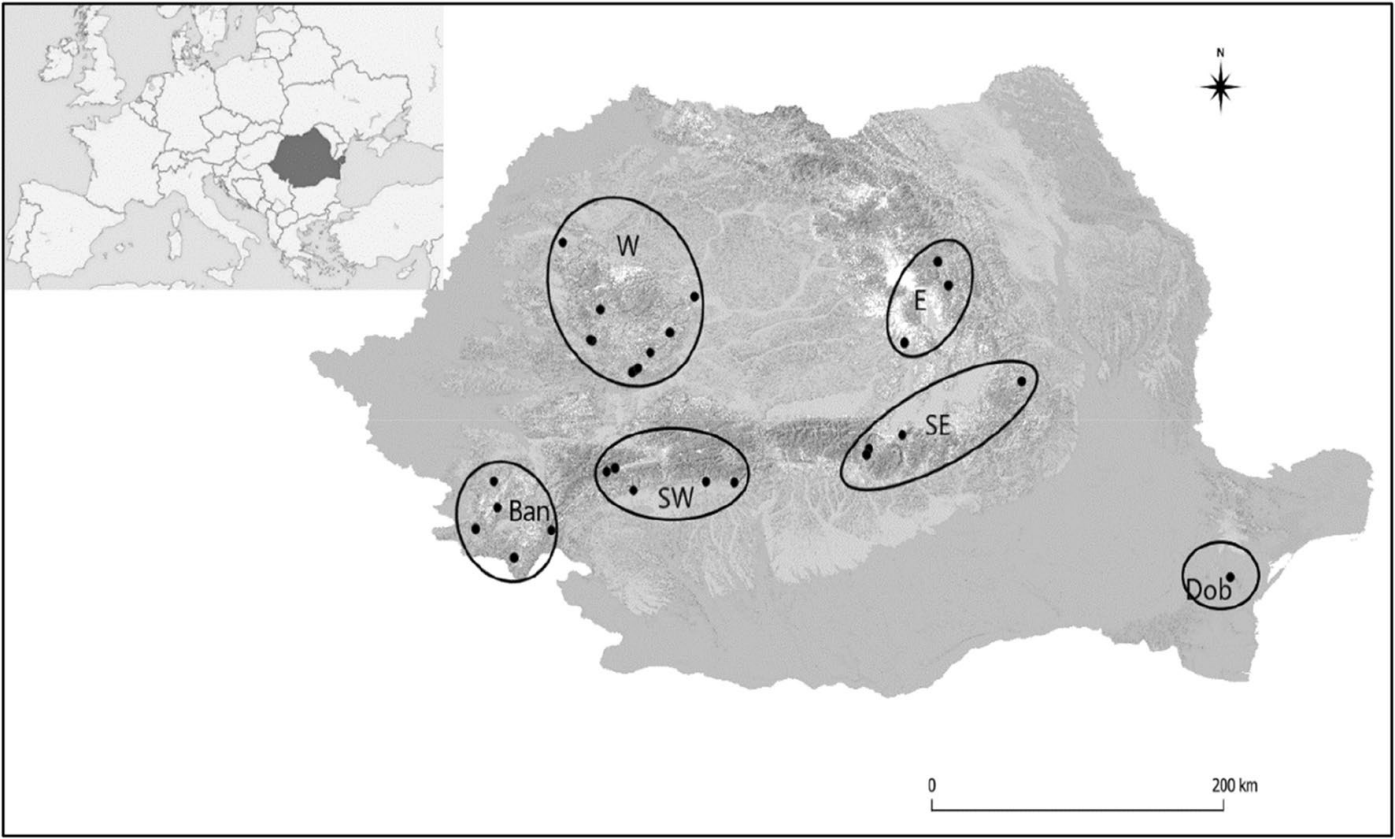
The location of the sampled gorges. The sampling points are divided into 6 groups according to their geographic location: W—West; Ban—Banat; SW—SouthWest; SE—SouthEast; E—East; Dob—Dobrogea. Details regarding the sampling areas are given in Appendix 1. The map was made in QGIS version 3.28 ^18^.

Samples were taken at 130 sites among 27 limestone gorges in the Romanian Carpathians and a further four in the Măcin Mountains, Dobrogea, between 2011 and 2019. The sampling points were located between 46.9742°, 44.6702° N, and 21.6931°, 28.4208° E. The altitude ranged between 34 and 1184 m. Figure 1 shows the location of the gorges, and full details of each site are given in Appendix 1.

In each sampling site, an area of about 100 m^2^ was sampled. Snails were searched for by two persons for one hour, following the procedures of Cameron and Pokryszko^19^. In addition, in each site, a 20 l sample of litter and topsoil was filtered through a 10 mm mesh sieve, and the resulting material was bagged and taken to the laboratory. The material was airdried, sieved and searched for snails directly or using a binocular microscope. To avoid bias due to shell accumulation, only live specimens and fresh empty shells with intact periostracum were considered in the analyses. While identification was generally to species, subspecies of the *Alopia* genus have been regarded as “species-level” taxa; their geographical distribution and conchological characters are distinct. Nomenclature generally follows Fauna Europaea; authorities are given in Appendix 2. Following the maps in Welter-Schultes^8^, each species was allocated to one of five geographical categories: 1, widespread in Europe; 2, Mainly Central and North-east Europe; 3, Balkan and South-east Europe; 4, mainly Carpathian; 5, restricted within the Carpathians.

At each sample site, the predominant characteristics of the habitat were recorded (Table 1). Altitude was also recorded, and tree cover was estimated by direct visual observation at each sample site. The climatic conditions for each site were obtained from bioclimatic data in WorldClim Version 2.1 (https://www.worldclim.org/) using the R Statistical Software R version 4.3.0^20^.

**Table 1:** Habitat characteristics recorded.

Habitat characteristics	Microhabitat	Dominant vegetation	Width of the gorge at the sampling point
Categories	1. Exposed limestone cliffs and crags 2. Limestone cliffs and crags in the forest 3. Large rocks in the forest 4. Forest floor with few rocks	1. Forest 2. Shrubs 3. Herbaceous vegetation	1. Narrow (limestone cliffs on both sides—distance under 70 m) 2. Intermediate (limestone cliffs on one side, or a 70–150 m distance) 3. Large (no limestone cliffs or distance over 150 m)

### Data analysis

There is great variation (77–3002; mean, 623.7; median, 424.5) in the number of specimens retrieved from each sample. While 29 samples fall below the suggested minimum ratio of ten specimens for each species overall^19^, the relationship between the number of specimens and species is not significant (r = 0.067 df 132, *p* > 0.05), abundance explaining less than 0.4% of the variation in richness. We have, therefore, avoided rarefaction.

We adopted a two-stage process for analysis. A presence and absence analysis involving all recorded species was used to ensure that characteristic but rare species were not ignored (see below). The species richness and composition were examined in relation to region, altitude, woody cover and dominant microhabitat. An unconstrained detrended correspondence analysis (DCA) was used to examine further the relationships among regions.

To include variation in abundance, we used multivariate analyses performed in Canoco 5.12 software^21^, transforming abundances by the expression y′ = log (y + 1). A preliminary analysis (DCA) including all species showed that the presence of many species in small numbers, often in only one sample, resulted in greatly extended ordination axes. To explore the response of species’ abundance to the considered factors, the linear constrained ordination—redundancy analysis (RDA)—is used. RDA may be applied when the gradient is short (species turnover is low), so we constrained the length of the axes, first eliminating the 22 species with fewer than six specimens recorded overall.

Samples from Dobrogea were also excluded, as they were very different from the others.

We classified our variables into two categories: those acting at large scale, climatic variables and geographic position (region) and those acting within gorges, features of the habitat. Given many correlations between climatic variables, we have chosen four to cover those that are easiest to interpret and encompass the major features of the climatic regime: bio1, mean annual temperature; bio4, temperature seasonality; bio12, annual precipitation; bio15, precipitation seasonality. There was a very close correlation (r = − 0.950) between altitude and bio1, and accordingly, altitude has been removed from the analyses.

We tested the significance of ordination axes by the Monte-Carlo permutation test with 999 unrestricted permutations per test. For the variables acting at large scale, the explained variation was calculated on the original data (by sample), but the significance of their effects was tested on the degrees of freedom given by the number of gorges to allow for possible pseudoreplication. To find the set of predictors that best explained species composition, we used interactive forward selection. Probabilities were adjusted to correct for the inflation of type-I error caused by multiple testing, using the false discovery rate values^22^. We evaluated the relative effects of the two groups of variables acting at large scale (region and climate) with variation partitioning. The effect of habitat variables was evaluated at both scales. The effect at the sample level (within the gorge) was tested in a partial RDA after partialling out the effect of the site (gorge). The effect at the site level (among gorges) was calculated by subtracting the variation explained at the sample level from the total variation explained by habitat variables.

Two post hoc analyses were carried out: on the influence of the speciose genus *Alopia* (Clausiliidae) on the results of RDA analyses and the qualitative and quantitative differences between *Cochlodina laminata* and *Granaria frumentum*, species with known differences in habitat preference.

## Results

### General features

Figure 1 shows the position of the 28 gorges. In the 134 sites examined, including those from Dobrogea, 133 species-level taxa were found among 83,718 shells. Appendix 1 lists the gorges and samples within each, their position and environmental features. Appendix 2 shows the species found at each site. There is a large tail of species encountered very rarely; excluding Dobrogea, eight are represented only by a single shell, and 22 are represented by fewer than six shells, singletons included. While we have restricted quantitative analyses based on numbers to those represented by at least six specimens (see Data analysis above), those uncommon species below this threshold may, nevertheless, reflect patterns of geographical and environmental differences. The mean number of species per site is 21.6 (SD, 6.25), and the median is 22; the range is 7–42.

### Presence and absence

Presence and absence analyses have been carried out for region (geographical variation), altitude (as a proxy for climate), woodland cover and predominant microhabitat (Appendix 3, Table S1). The species unique to each category and the numbers involved are listed in Appendix 3, Table S2.

Species richness varies greatly within all categories in each analysis, indicating that these categories do not account for all or even most of this variation, nor does it relate to any variation in mean sample size. Nevertheless, and without formal statistical analysis, there are patterns of variation both in species richness and the proportion of species unique to each category. Among regions, excluding Dobrogea, there is more variation in richness and a greater proportion of species unique to each than in the habitat categories other than altitude. Altitude and region are correlated, with samples below 300 m being almost exclusive to the Banat, while SouthEast contains most of the high-altitude samples. Each region and altitude class holds a smaller proportion of the total species richness than any of the categories used to describe the habitat.

While most samples were made in a forested environment, the predominant habitat at the site varied, as shown by the amount of woody cover. There is a correlation between this and microhabitat; exposed limestone cliffs have little tree cover. When unique species are examined (Appendix 3, Table S2, S3), it is apparent that a greater proportion of these are represented by species recorded in very low numbers in these habitat categories than in those for region and altitude. The larger proportion of abundant unique species among regions and with respect to altitude is influenced by the known and extremely limited distribution of species in certain genera. Among these, taxa in the clausiliid genus *Alopia* have a major effect on the numbers involved (Appendix 3 Table S2). All 16 taxa are restricted to single regions, and 10 are confined to a single altitude class. Only five are restricted among both canopy cover and principal categories used to describe the habitat. By contrast, the reverse pattern is seen in species that are found in all categories in each analysis; less than 20% of the fauna is common to all regions, but more than twice as much in categories of woody cover and microhabitat.

**Figure 2:**
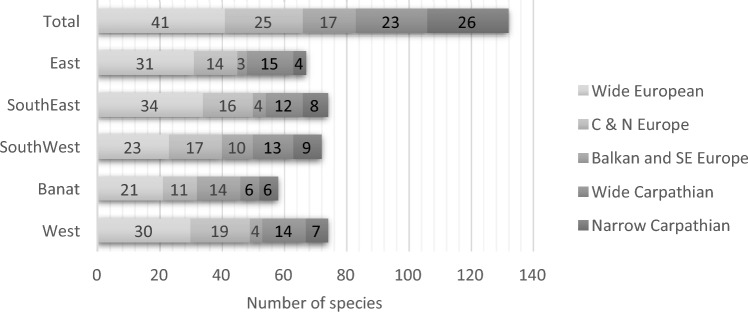
Number of species in each region considered by geographical range.

In the overall fauna recorded (Appendix 2), 50% of species have wide or central European distributions, 13% are Balkan or Southern European, and 37% have a Carpathian distribution in the broad sense. There are differences between regions in the balance of species so classified (Fig. 2). Apart from Dobrogea, Banat stands out for the high proportion of Balkan and SE European species, while SouthWest, nearby, is intermediate in this respect from the remainder. Both have a lower proportion of broad-range species than the rest.

The four samples from a single gorge in Dobrogea are distinctive in being species-poor despite very large sample sizes. Five of the 19 species found do not occur in any of the other regions. The affinity of these faunas is more with those from the Banat than any others, despite the great distance between them. Thus, 14 species (all those not unique to Dobrogea) are in common with Banat, as opposed to eight or nine for other regions.

### Quantitative analyses

**Figure 3:**
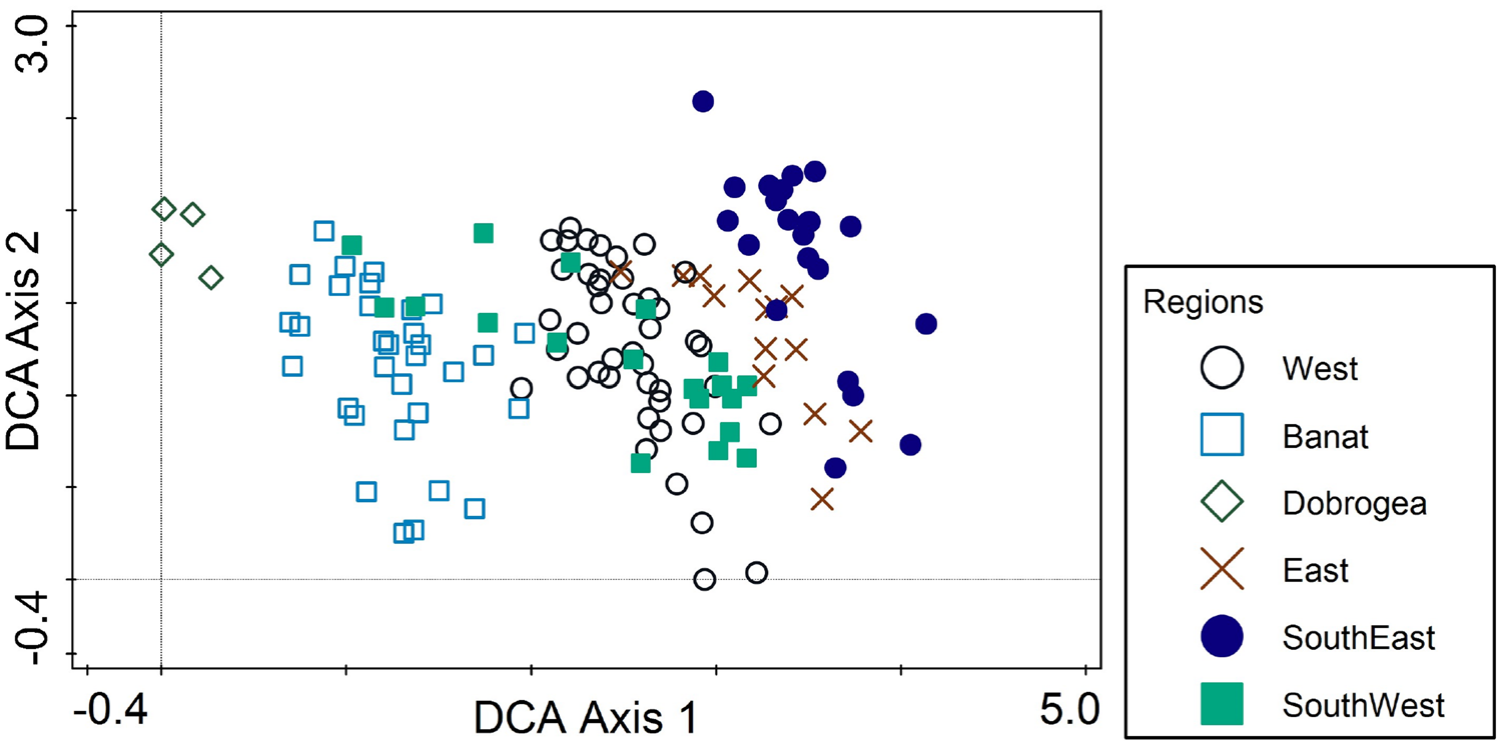
Sample distribution on the first two axes of an unconstrained DCA analysis based on the presence or absence of all species.

The distinctive fauna of each region is confirmed by an unconstrained DCA analysis based on presence and absence data (Fig. 3). While there is overlap, samples cluster by region on the first axis, which extracts 8.3% of the variation. While there is an east-to-west trend in the Carpathians proper, it is notable that the sites from Dobrogea are closest to those from Banat, the most distant region. Both contain mainly low-altitude and southern sites. The second axis (3.4% of the variation) does not separate regions with any clarity.

**Figure 4:**
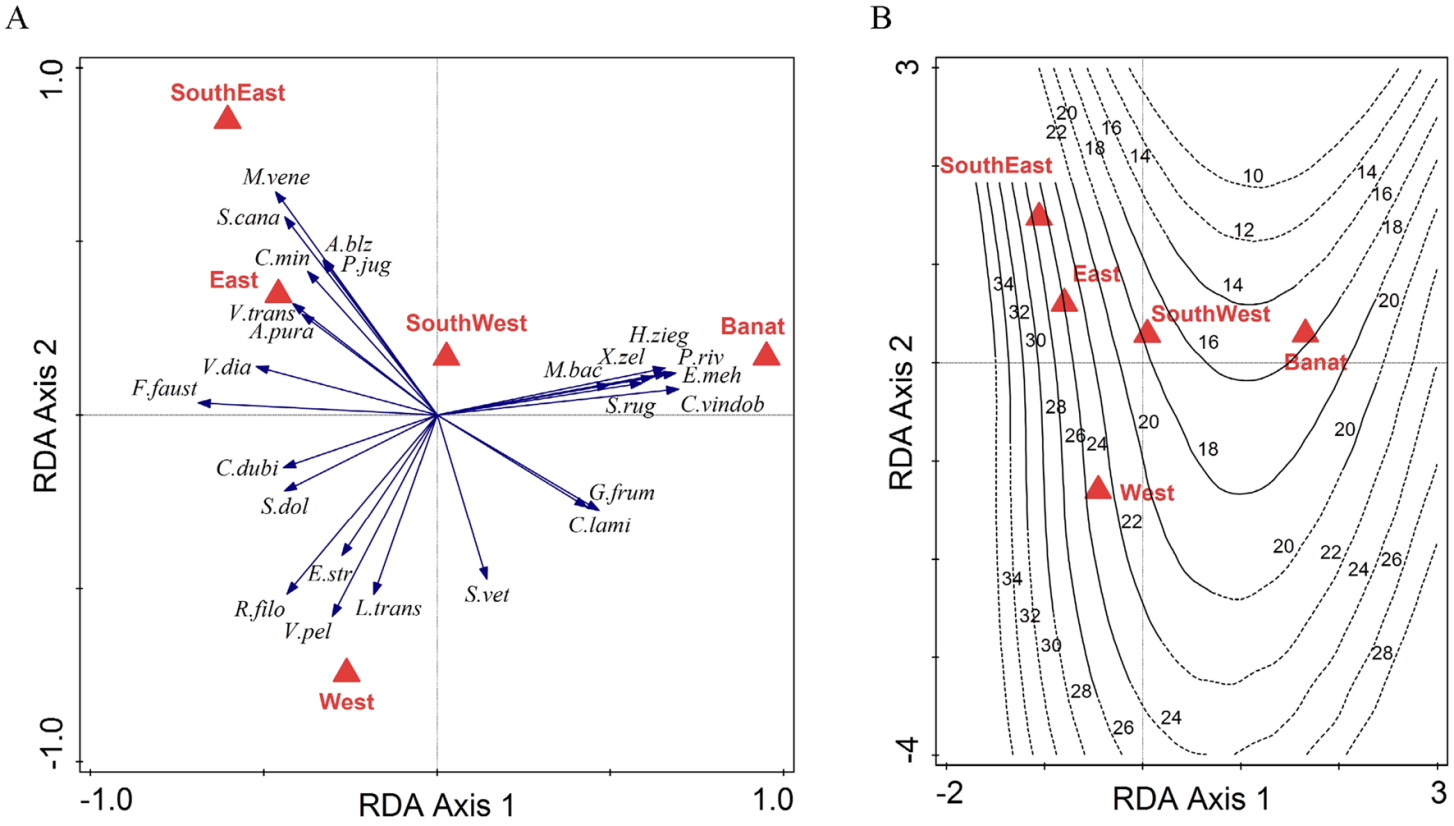
RDA ordination plots illustrating A. the position of the 25 best fitting species in relation to the geographic regions in the ordination space defined by the first two constrained axes, B. the isolines of species richness in the same space. Species abbreviations are given in Appendix 2.

In RDA analyses, regions and climate account for similar proportions of variance (regions—25%, 22.6% adjusted, pseudo-F = 10.4, *p* = 0.001; climate—23.9%, 21.4% adjusted, pseudo-F = 9.8, *p* = 0.001) with the first two axes accounting for about half of that explained (Table 2). For regions (Fig. 4A), the first axis (explained variation, 49.1%, pseudo-F = 4.4, *p* = 0.001) separates Banat from the rest, by having high abundances of *Pomatias rivularis, Euomphalia mehadiae, Herilla ziegleri, Caucasotachea vindobonensis, Xerocampylaea zelebori, Monachoides bacescui, Strigillaria rugicollis,* which represent a distinct suite associated strongly with Banat*.* The second axis (accounting for 31.2% of the explained variation) contrasts SouthEast, which has high abundances of *Mastus venerabilis, Strigillaria cana, Argna bielzi, Carichium minimum, Pseudalinda jugularis*, with West, where *Vitrina pellucida, Lozekia transsylvanica, Ruthenica filograna, Euomphalia strigella* prevail. SouthWest is the most similar to Banat. Species richness increases from west to east and slightly from north to south, being minimum in Banat and maximum in SouthEast (Fig. 4B). In the loess model, the geographic region explains 62.7% of the variation in species richness.

**Table 2:** Simple and conditional term effects of predictors in the three redundancy analyses (RDA) relating the land snail species abundance to the geographic regions (Range), climatic variables (Climate) and habitat characteristics (Habitat). P(adj) represents p-values corrected for false discovery rates.

Range						
Simple term effects				Conditional term effects		
Predictor	Explains %	pseudo-F	P(adj)	Explains %	pseudo-F	P(adj)
area.Ban	11.4	16.5	0.001	11.4	16.5	0.001
area.West	8.1	11.2	0.001	7.2	11.3	0.001
area.SouthW	2.7	3.6	0.001	4	6.5	0.001
area.SouthE	7	9.6	0.001	2.4	3.9	0.001
area.East	3.2	4.2	0.001			
Total explained variation (adjusted) %						25 (22.6)

Climate						
Simple term effects				Conditional term effects		
Predictor	Explains %	pseudo-F	P(adj)	Explains %	pseudo-F	P(adj)
bio15 (precipitation seasonality)	11.2	16.2	0.001	11.2	16.2	0.001
bio1 (mean annual temperature)	10.7	15.4	0.001	6.8	10.5	0.001
bio12 (annual precipitation)	5.1	6.8	0.001	2.4	3.9	0.001
bio4 (temperature seasonality)	2.6	3.4	0.001	3.5	5.6	0.001
Total explained variation (adjusted) %						23.9 (21.4)

Habitat						
Simple term effects				Conditional term effects		
Predictor	Explains %	pseudo-F	P(adj)	Explains %	pseudo-F	P(adj)
width	4	5.4	0.001	4	5.4	0.004
microhabitat	3.6	4.8	0.001	4	5.5	0.004
aspect.S	3.7	5	0.001	1.8	2.4	0.004
tree cover	3.6	4.8	0.001	1.2	1.7	0.063
vegetation	2.3	3.1	0.001	1.2	1.6	0.077
aspect.N	1.6	2.1	0.009	0.6	0.8	0.691
aspect.E	1	1.3	0.147	0.8	1.1	0.347
aspect.W	0.6	0.8	0.71			
Total explained variation (adjusted) %						13.5 (8.5)

**Figure 5:**
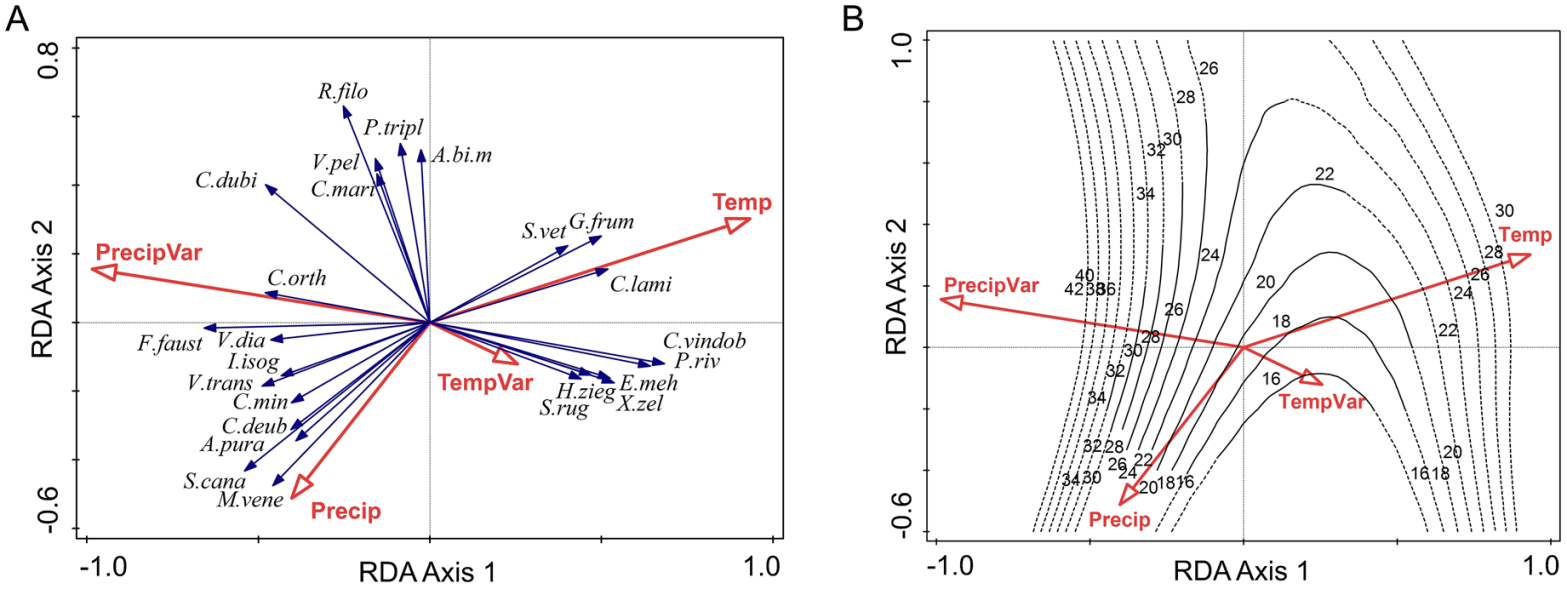
RDA ordination plots illustrating A. the position of the 25 best fitting species in relation to the climate variables in the ordination space defined by the first two constrained axes, B. the isolines of species richness in the same space. Temp—mean annual temperature, TempVar—temperature seasonality, Precip—annual precipitations, PrecipVar—precipitation seasonality. Species abbreviations are given in Appendix 2.

A similar pattern emerges for climate (Fig. 5A), where the first axis (explained variation, 47.6%, pseudo-F = 4, *p* = 0.001) contrasts the effect of temperature (annual mean value—Temp and seasonality—TempVar) and precipitation (annual mean value—Precip and seasonality—PrecipVar), while the second axis (accounting for 34.5% of the explained variation) is given by the opposition between annual values and seasonality. Precipitation seasonality and mean annual temperature are the best climatic predictors of snail community structure, explaining similar amounts in its variation (11.2% and 10.7%, Table 2). The same suite of species characteristic for Banat is associated with high temperature seasonality and low precipitation seasonality. More generally, the pattern of species loading is very similar in both analyses; most of the species that show a strong geographical response also have strong responses to climate. Species richness increases sharply with precipitation seasonality and slightly with annual values of precipitation and temperature, while temperature seasonality has a slight negative effect (Fig. 5B). In the loess model, the climate variables explain 62.8% of the variation in species richness.

**Figure 6:**
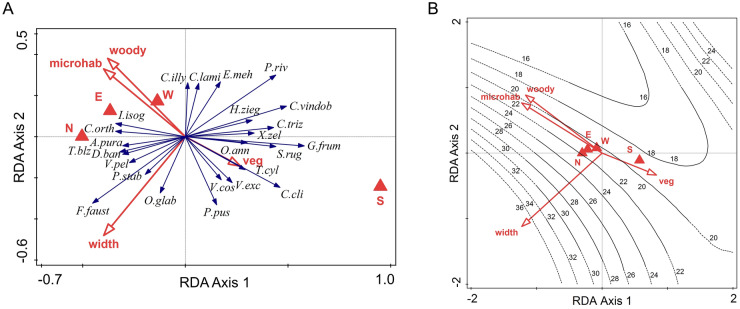
RDA ordination plots illustrating (**A**). the position of the 25 best fitting species in relation to the habitat features in the ordination space defined by the first two constrained axes, (**B**). the isolines of species richness in the same space. N, S, E, W—slope aspect, veg—vegetation cover, woody—woody vegetation cover, width—width of the valley, microhab—microhabitat type. Species abbreviations are given in Appendix 2.

Habitat factors account for less than half as much variation in species composition (13.5%, 8.5% adjusted, pseudo-F = 2.7, p = 0.001) as region and climate, despite the number of factors considered (Tables 1, 2; Fig. 6A). Here, however, the loading of species relates clearly to what is known of their ecology, essentially forest species (*Drobacia banatica, Trochulus bielzi, Aegopinella pura, Vitrina pellucida, Cochlodina orthostoma, Isognomostoma isognostomos, Pseudalinda stabilis*) being positively associated with forest floors with small rocks in old forests with northern aspect, high woody vegetation cover, while *Granaria frummentum, S. rugicollis, Chondrina clienta, C. vindobonensis, Campylaea trizona* prevail in herbaceous vegetation on southern slopes. Species richness increases sharply with the gorge width and is relatively independent of the other numerical habitat features. South-facing slopes have a somewhat lower number of species compared to other cardinal directions (Fig. 6B). In the loess model, the habitat features explain 48.8% of the variation in species richness.

**Figure 7:**
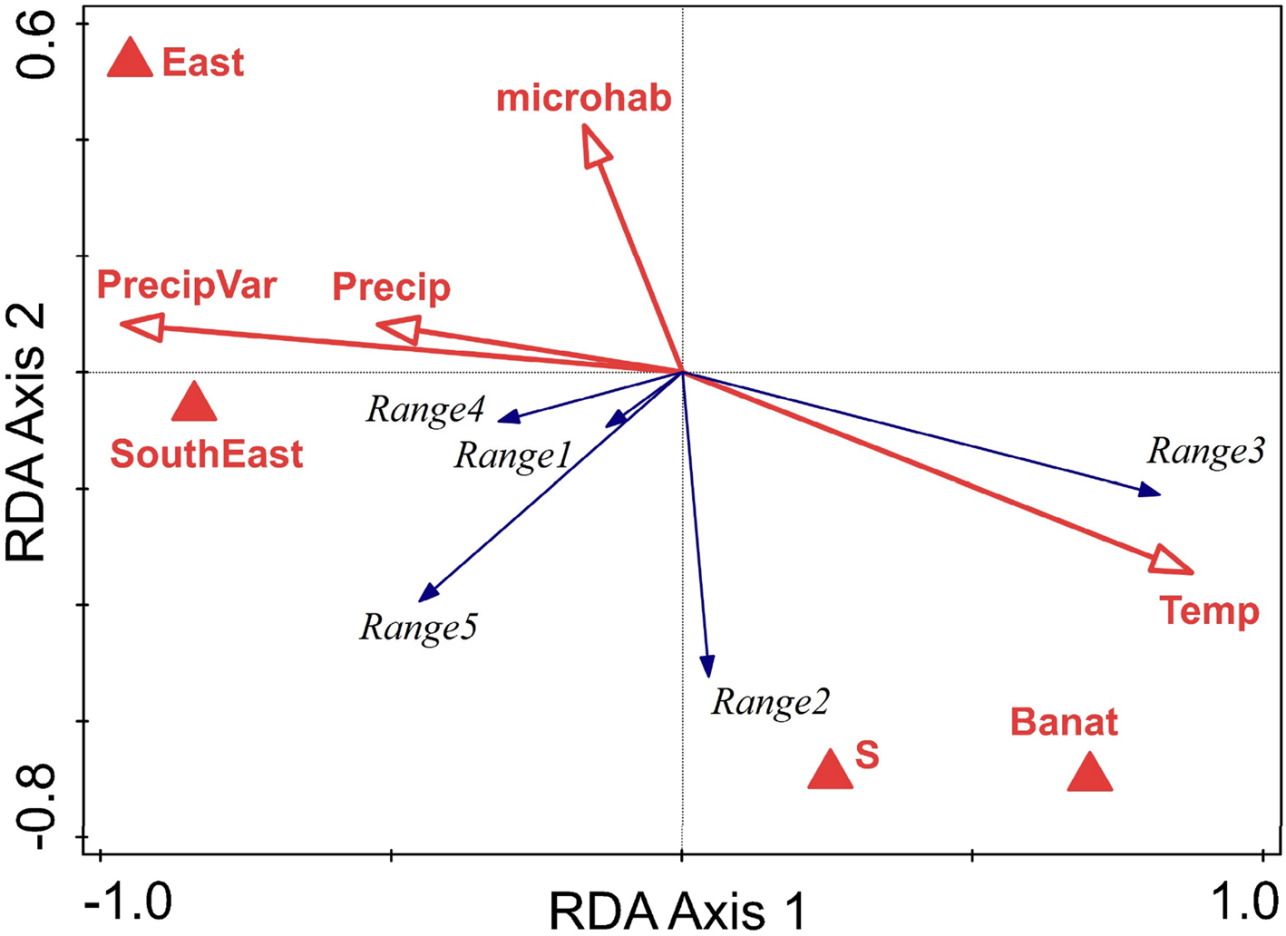
Biplot of region, climate and habitat factors in relation to species aggregated by geographical range in an RDA ordination. Range 1—European widespread; Range 2—Central European; Range 3—Balkan and SE. Europe; Range 4—Mainly Carpathian; Range 5—Restricted Carpathian. Temp—mean annual temperature, Precip—annual precipitations, PrecipVar—precipitation seasonality, S—southern slope aspect, microhab—microhabitat.

The best model of the snail community structure in terms of categories of species aggregated by geographical range included Banat, East and SouthEast regions, S slopes, Temp, Precip, PrecipVar, and microhabitat, which explained 46.6% (43.2% adjusted, pseudo-F = 13.6, *p* = 0.001) of the variation in category composition (Fig. 7). The first axis (accounting for 58.2% of the explained variation) is given mainly by the climatic variables and the opposition between eastern and western (Banat) regions. A clear connection can be seen between species of southern and Balkan ranges (Range3) with warm, dry climate. Species with Central European distribution (Range2) are associated with South-facing limestone rocks, while Carpathian species (Range4 and Range5) appear aligned with the dry eastern and mostly southeastern regions. Species with wide, European distribution (Range1) show the weakest response to the region, climate and habitat.

**Figure 8:**
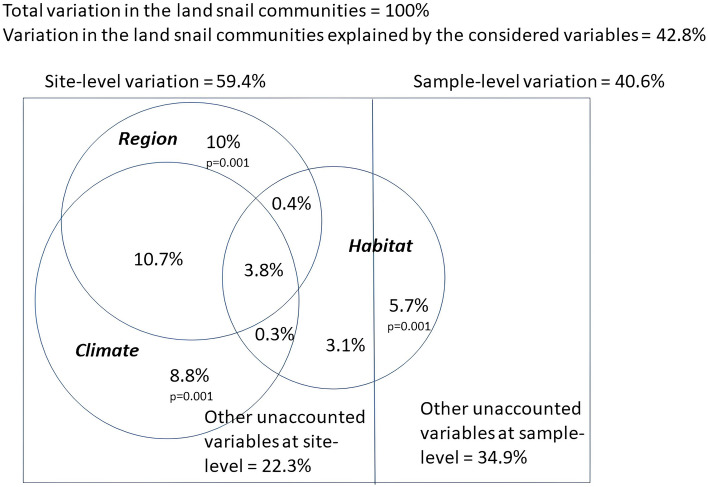
Partition of variation in snail community composition explained by the factors acting at the site (gorges) and sample level.

When variance is partitioned according to the factors examined (Fig. 8), their interrelationships are revealed. Overall, 42.8% of the variation is accounted for by all the variables considered. However, most of the variation in snail species composition (59.4%) is among sites (gorges), where the unique effect of region (10%) is about the same as that of climate (8.8%) and more than double that of the geographic position. As expected, the overlap between region and climate is the largest (10.7%). The lesser effect of habitat is apparent. Habitat variables affect community composition both at site and sample level (among and within gorges), having a slightly stronger effect at site level (7.6%), while within gorges, they account for only 5.7% of total variance. The variation unaccounted for by the considered variables is higher at the sample level than at the site level (34.9% compared to 22.3%).

### Post hoc analyses

As demonstrated above, the patterns revealed here are a product of the different responses of species to the variables considered. An extreme example is that of *Alopia* species, all of which show very restricted ranges and which replace each other among samples even within the same region. With 16 species-level taxa, about 14% of all those considered in the quantitative analyses, this single genus alone could influence the results. Table 3 shows that this is not the case; the pattern of variation in the data remains much the same whether *Alopia* is omitted or if all members of the genus are regarded as a single species.

**Table 3:** The variance accounted for in RDA (%) when *Alopia* species are combined or omitted from the overall analysis as given in Table 2.

Analysis	Regions	Climate	Habitat
As Table 2	22.5	21.4	8.5
*Alopia* combined	23.5	22.5	8.5
No *Alopia*	23.2	21.9	8.9

The complex relationship between climate, location and habitat can be illustrated by the distribution and abundance of two geographically widespread species, *Cochlodina laminata* and *Granaria frumentum.* While they are positively associated among samples on a presence and absence basis (χ^2^ = 10.1, *P* < 0.01), when abundance is considered (Table 4, Fig. 4A, 6A), there is a clear difference: While the distributions among regions and aspects are similar, and account for their loadings in the RDA for regions, they differ in the associated features of predominant microhabitat and tree cover. The shared preference for low, warm sites contrasts with the divergent tolerance of differing amounts of cover.

**Table 4:** The % of samples in each category of Region, Aspect, Microhabitat and Tree Cover in which *Granaria frumentum* and *Cochlodina laminata* occur in numbers greater than the median (52 and 5 respectively) among samples in which they were recorded.

Region	%	Aspect	%	Microhabitat	%	Tree cover %	%
*Granaria frumentum*							
Banat	58	East	50	1 (exposed limestone cliffs )	65.78	0–10	75
West	56	North	23.8	2 (limestone cliffs in the forest)	75	15–30	66.66
South West	50	South	63.33	3 (large rocks in the forest)	38.88	40–60	81.25
South East	16.66	West	55.55	4 (few rocks in the forest)	21.42	> 70	26.47
East	33.33						
*Cochlodina laminata*							
Banat	60.71	East	42.8	1 (exposed limestone cliffs)	50	0–10	16.66
West	53.57	North	47.5	2 (limestone cliffs in the forest)	46.15	15–30	40
South West	33.33	South	52.38	3 (large rocks in the forest)	55.55	40–60	58.82
South East	0	West	27.27	4 (few rocks in the forest)	33.33	> 70	47.36
East	25						

## Discussion

The primary conclusion of our study is that differences in the land snail faunas of these Carpathian limestone gorges are determined more by location, climate (which is strongly influenced by altitude), or a combination of the two, than by habitat factors such as the extent of woody cover or the microhabitat characteristics at each site. This pattern is reflected in both the presence and absence analyses and in the RDA. It is reinforced by the greater proportion of species unique to single categories of region or altitude than to any habitat category and their occurrence in numbers that lower the chance of mere sampling accidents accounting for their absence elsewhere. Most species unique to any habitat category are very uncommon both in frequency of occurrence and numbers found. Species richness also varies among regions, and the observed range of richness among samples in each region varies greatly.

Separating the effects of location, habitat and climate/altitude is not straightforward. The Banat snail fauna is clearly separated from those of the other regions by the first axis of the RDA diagram and by the presence and absence analysis. The distinct character of the malacofauna of this region was shown by previous studies^12^ and is also recorded for other invertebrate^23,24^ and vertebrate groups^25–27^. These samples, however, constitute nearly all those at the lowest altitude and those subject to a dry, warm climate. They are also relatively species-poor, as is the fauna of the region in our study. Although they contain a higher proportion of species with Balkan and SE European affinities than other regions, as also noted by Cameron et al.^12^, they hold more species in common with those from Dobrogea, far distant, but at low altitude and lowest latitude. The findings are consistent with the extent of the Submediterranean climate in Romania, as described by several authors^28–30^. Two areas are considered to have a Submediterranean climate, one in the south-west of Romania, between Sânnicolau Mare and Drobeta Turnu Severin, described as Dacian-type Submediterranean climate by Ghibedea & Isbăşoiu^29^, and the second in Dobrogea and Zimnicea-Giurgiu sector^30^. These areas are characterized by shorter and warmer winters, usually associated with rainfall and sleet, due to the south-west air advections generated by the Mediterranean cyclones^30^. The precipitation regime of the Submediterranean climate is intermediate between the continental and Mediterranean climate, with two pluviometric maxima in May and June (like the continental climate) and a secondary one in October–December, as in the Mediterranean climate^29^.

These particular climatic features, accentuated by sampling on limestone, affect the characteristics of flora and fauna^31–34^, admitting not only species with a Balcanic distribution but also those from Dobrogea containing Pontic elements. Nevertheless, very local variations in microclimate and soils can alter the picture. Three samples made by Cameron et al.^12^ in the Banat were both much higher (c. 700 m asl) and on schist rather than on limestone. Nine species were found only in these three sites (Table 5). All are missing from the Banat samples reported here, but seven were found in other regions. There is a mix of species typical of Carpathian forests and those found also in forests far to the north in Europe. No species in these sites is associated with rocks, and none have Balkan or SE European affinities, whereas these were most frequent in samples from the Banat in this study.

**Table 5:** Species recorded only among three sites on high altitude forest on schist in the Banat by Cameron et al. ^12^, and their presence or absence in the Banat and other regions surveyed in this study.

Species	Banat ^12^	Banat (this study)	Elsewhere (this study)
*Pseudalinda fallax*	+	−	+
Pseudalinda stabilis*	+	−	+
*Clausilia dubia*	+	−	+
*Vestia turgida*	+	−	+
*Carychium minimum*	+	−	+
*Cochlicopa lubrica*	+	−	+
*Eucobresia nivalis*	+	−	−
*Vertigo substriata*	+	−	−
*Zonitoides nitidus*	+	−	+
*Arianta arbustorum*	+	−	+

*Pseudalinda stabilis was also recorded from one high-altitude site on limestone.

The samples from the SouthEast, mostly at high altitudes, are the most distinct from those from Banat, as shown both in the presence and absence analysis and the RDA. It has the largest number of unique species, seven of the 16 *Alopia* taxa being found here (the remaining *Alopia* taxa are equally distributed, respectively three species each, between the West, SouthWest and East regions). This genus is endemic to the Romanian Carpathians, with the exception of a Slovakian subspecies. The eastern part of the Southern Carpathians represents its center of diversity^35–38^, where the genus is represented by numerous, often polytypic species with high morphological variation. Species of *Alopia* show a strong preference for mostly rocky limestone habitats. We note, however, that removal of this genus from the analysis, or regarding all members as a single taxon, does not significantly affect the overall partition of variance; this is not a pattern produced by a single genus.

The overall species richness is best explained by climatic parameters, with taxonomic diversity increasing with precipitation seasonality and mean annual temperature. As concluded by many studies, the main limiting factors of snail distribution in terrestrial ecosystems, are calcium availability and moisture^39–44^. If the first one is secured on limestone, the second one largely depends on microhabitat characteristics but mostly on precipitation amount and temperature, both parameters with a strong seasonal character. Seasonality is the most omnipresent source of external features influencing natural systems^45,46^. A series of factors with seasonal variation represents drivers of selection (temperature, precipitation, food resources); therefore, they are crucial for species diversity^46^. There are studies confirming the role of the seasonal character of different climatic factors on biodiversity. Tonkin et al.^47^ found an increased diversity with seasonality in stream invertebrate communities. For freshwater invertebrates, the species diversity seemed to be generated by a balance between seasonality (rainfall and temperature) and predictability. Similarly, precipitation seasonality explained best the increase in the diversity of freshwater snails^48^. Other studies are showing comparable results for terrestrial habitats. Thus, precipitation seasonality was singled as the best predictor of soil invertebrate richness in agricultural ecosystems in China^49^, while, in urban habitats, snail abundance was negatively associated with increasing rainfall and positively but weakly associated with higher temperatures during the winter^50^. The effect of precipitation seasonality on species richness, in our case, could be just another way to capture the effect of Submediterranean climate, characterized by a lower seasonality of precipitation. On the other hand, the role of increased mean annual temperature in species richness for land snails was also confirmed by several studies^16,44,51^.

The pattern of species richness could be as well the expression of the role of different Carpathian areas as glacial refugia. Although we tend to refer to a single ‘Carpathian refugium’, most probably, different parts of the mountain range often acted as distinct, isolated refugia^1,4,6^, with the most climatically stable and largest areas in the Southern Carpathians^52^. In some cases, some snail species underwent long-term isolation and eventual speciation, as in the case of other groups^1,6^. We note that more species are unique to exposed limestone cliffs than to forested sites; while sampling error may account for some of this difference, limestone cliffs have, perhaps, been subject to more frequent and prolonged periods of isolation through glacial cycles.

Although the importance of physical habitat characteristics for land snails is well documented^53–57^, in our study, they appear to play a smaller part in determining composition and richness than region and climate. Nevertheless, there is evidence of their effects, both in the RDA analyses and when individual comparisons are made, as between *C. laminata* and *G. frumentum* shown above. Species associated with warm, dry, insolated habitats are contrasted with those associated with cool, damp and shaded habitats. Such differences, however, do not relate directly to geographical range: there are widespread and narrow-range species in each category.

In the context of the relationship between regional and local diversity and composition, our results suggest that such relationships are complicated by the difficulty in defining region for a fauna in which some species show evidence of either vicariance or extremely limited powers of dispersal, while others have dispersed widely after the last glacial period. The Carpathians are a known refugium for forest species in the Pleistocene^58^. While Hausdorf & Hennig^59^ concluded that the snail fauna of north-west Europe was shaped mainly by rapid post-glacial dispersal, this is not the case here. Scale matters^60^, but as elsewhere, even within the same genus, there are those with extensive ranges and others showing very local endemism; the ecology of such differences is largely unexplored^61^.

### Supplementary Information

Supplementary Information 1.

Supplementary Information 2.

Supplementary Information 3.

## Data Availability

All data generated or analysed during this study are included in this published article and its supplementary information files.
