# Supplementary material for: Geographical and environmental patterns of Carpathian land snail faunas in a region of high endemicity
Source: Sci Rep. 2024 Jan 16;14:1392. doi: 10.1038/s41598-024-51870-6 (PMC10791649; doi:10.1038/s41598-024-51870-6)
Supplement: Supplementary file 1 — Supplementary Information 1. [file 41598_2024_51870_MOESM1_ESM.pdf]

Geographical and environmental patterns of Carpathian land snail faunas in a region of high endemicity

VOICHIȚA GHEOCA, ANA MARIA BENEDEK, ROBERT CAMERON

Appendix 1.

Site details including geographic location, coordinates, altitude range, and number of samples in each site.

No	Sampling area	Abbrev	Number of samples	Geogr. location	Latitude	Longitude	Altitude (m)
1	Pădurea Craiului Mountains – Crișul Repede Gorge	PC	7	West	46.9615 -46.9741	22.5083 - 22.5168	290-321
2	Trascău Mountains – Turda Gorge	TD	5	West	46.5644-46.5673	23.6720-23.6794	411-465
3	Trascău Mountains - Râmeț Gorge	RAM	4	West	46.2948- 46.2959	23.4626- 23.4672	843-849
4	Trascău Mountains – Feneș Gorge	FE	2	West	46.15211-46.1526	23.2858-23.2859	537-540
5	Bihorului Mountains- Ordâncușa Gorge	O	4	West	46.4631-46.4750	22.8385-22.8477	753-787
6	Metaliferi Mountains – Ardeu Gorge	A	4	West	46.0173- 46.0328	23.1411- 23.1459	416-481
7	Metaliferi Mountains – Mada Gorge	M	5	West	46.0074- 46.0075	23.1250- 23.1258	348-355
8	Metaliferi Mountains – Cib Gorge	CI	4	West	46.0217- 46.0363	23.1719- 23.1782	382-526
9	Zaranului Mountains – Ribicioara Gorge	RIB	5	West	46.2139- 46.2384	22.7617- 22.7741	313-350
10	Zarandului Mountains – Bulzești limestones	BULZ	3	West	46.2461- 46.2936	22.7540- 22.7553	419-539
11	Anina Mountains – Caraș Gorge	CA	6	Banat	45.2019- 45.2103	21.8754- 21.9016	216-275

12	Anina Mountains – Miniș Gorge	MI	3	Banat	45.0126- 45.0131	21.9213- 21.9333	421-440
13	Anina Mountains – Nera Gorge	NE	5	Banat	44.8561- 44.9031	21.7364-21.7489	198-330
14	Anina Mountains – Pecinişca Gorge	P	3	Banat	44.8445-44.8567	22.4102-22.4121	241-292
15	Banatului Mountains – Iron Gates	PF	15	Banat	44.6053-44.6701	21.6995-22.2901	83-251
16	Retezat Mountains – Buta Gorge	BUT	2	SouthW	45.30204-45.3031	22.9734-22.9744	950-952
17	Retezat Mountains – Scorota Gorge	SCOR	2	SouthW	45.2760-45.2769	22.8996-22.9003	1054-1184
18	Vâlcan Mountains – Sohodol Gorge	SOH	5	SouthW	45.1352- 45.1398	23.1353- 23.1429	450-485
19	Parâng Mountains – Olteţului Gorge	OLT	5	SouthW	45.1388- 45.3010	23.7660- 23.7889	622-784
20	Căpăţânii Mountains – Bistriţa Gorge	BIS	5	SouthW	45.1912- 45.1973	24.0307 24.0359	665-697
21	Piatra Craiului Mountains – Dâmbovicioara Gorge	DAMB	6	SouthE	45.4180- 45.4547	25.2085- 25.2223	876-986
22	Piatra Craiului Mountains – Podul Dâmboviţei	PD	4	SouthE	45.3985- 45.4014	25.2009- 25.2024	830-832
23	Piatra Craiului Mountains – Zărneşti Gorge	ZAR	7	SouthE	45.5233- 45.5460	25.2168- 25.2698	886-1129
24	Ciucaș Mountains – Valea Stânii Gorge	CIUC	5	SouthE	45.4982- 46.6452	25.9264- 26.0072	990-1140
25	Vrancei Mountains – Tișița Gorge	TIS	3	East	45.9113- 45.9401	26.5532- 26.5739	635-763
26	Perşani Mountains – Vârghiș Gorge	VG	4	East	46.2209- 46.2235	25.5368- 25.5436	530-558
27	Hăşmaș Mountains – Bicaz Gorge	BIC	7	East	46.8064- 46.8206	25.8108- 25.8390	763-1043
28	Dobrogea Gorge	DOB	4	Dobrogea	44.5013- 44.5058	28.4208- 28.4291	34-55
