# Supplementary material for: Geographical and environmental patterns of Carpathian land snail faunas in a region of high endemicity
Source: Sci Rep. 2024 Jan 16;14:1392. doi: 10.1038/s41598-024-51870-6 (PMC10791649; doi:10.1038/s41598-024-51870-6)
Supplement: Supplementary file 3 — Supplementary Information 3. [file 41598_2024_51870_MOESM3_ESM.pdf]

Geographical and environmental patterns of Carpathian land snail faunas in a region of high endemicity

VOICHIȚA GHEOCA, ANA MARIA BENEDEK, ROBERT CAMERON

Appendix 3.

Table S1. Features of the samples as segregated by categories of region, altitude, woody cover and microhabitat. % of total fauna refers to that excluding Dobrogea. % unique relates to the number of species in that category. *, for species found only in Banat and Dobrogea (see text). N represents the number of samples included in each category.

**Region**	**N**	**Median and range richness**	**Median and range, altitude, m.**	**Median and range % Woody cover**	**Median and range, Specimens**	**Total species**	**% of total fauna**	**% Unique**
**West**	43	22.0<br>12-38	419<br>290-849	60<br>10-100	627<br>93-3002	74	56.1	13.5
**Banat**	32	17.0<br>7-29	242.5<br>83-292	50<br>5-90	467<br>76-2388	58	43.9	17.2<br>*20.7
**South W**	19	22.0<br>12-30	622<br>450-1184	50<br>5-100	251<br>95-1428	72	54.5	13.9
**South E**	22	26.5<br>12-42	983<br>830-1140	30<br>5-100	315.5<br>90-857	74	56.1	14.9
**East**	14	25<br>14-37	763<br>551-1043	80<br>0-100	259<br>88-1011	67	50.8	7.5
Dobrogea	*4*	*14.0*<br>*11-17*	*48*<br>*34-55*	*10*<br>*0-40*	*1154*<br>*606-2006*	*19*	*14.2*	*26.3*

**Altitude class, m**	**N**	**Median and range richness**	**Median and range % Woody cover**	**Median and range, Specimens**	**Total species**	**% of total fauna**	**% Unique**
**<300**	28	17.5<br>7-29	55<br>5-90	467<br>76-2388	58	43.2	12.1
**300-500**	39	22.0<br>12-32	60<br>5-100	765<br>133-3002	78	59.1	7.7
**500-700**	21	22.0<br>14-38	80<br>5-100	259<br>95-2700	80	60.6	10.0
**700-900**	19	27.0<br>17-33	40<br>5-100	273<br>133-627	83	62.9	7.2
**>900**	23	23.0<br>12-42	70<br>0-100	249<br>90-857	83	62.9	10.8

**Woody cover class**	**n**	**Median and range richness**	**Median and range Altitude, m**	**Median and range, Specimens**	**Total species**	**% of total fauna**	**% Unique**
**0-10%**	21	21<br>7-27	485<br>83-1140	749<br>76-2229	91	68.9	6.6
**15-30%**	25	22.0<br>9-42	538<br>102-1140	466<br>133-1994	97	73.5	4.1
**40-60%**	24	22.0<br>12-29	417<br>87-1140	541<br>111-2700	89	67.4	2.2

**70-80%**	36	23.0<br>12-37	454<br>106-1082	377<br>93-3002	103	78.0	2.9
**80-100%**	24	21<br>12-38	590<br>128-1184	246<br>88-1379	95	72.0	5.3
**Micro-habitat**	**n**	**Median and range richness**	**Median and range Altitude, m**	**Median and range, Specimens**	**Total species**	**% of total fauna**	**% Unique**
**Exposed cliff**	53	21<br>7-33	475<br>83-1140	534<br>76-2388	111	84.1	9.0
**Shaded cliff**	22	22.5<br>12-42	495.5<br>98-1140	440.5<br>90-3002	100	75.8	5.0
**Forest large rocks**	30	23.0<br>12-38	544<br>115-1184	317<br>88-2048	97	73.5	3.1
**Forest small rocks**	25	21.0<br>12-37	530<br>142-1054	243<br>93-1379	94	71.2	3.2

Table S2. Species confined to single categories, and the numbers recorded for each. For all categories, species found only in one category, but also in Dobrogea are marked *. They are regarded as being unique within the context of the Carpathians (see text)

**REGIONS**					
**West**		**South West**		**Banat**	
Alopia bielzi madensis	1451	*Alopia vicina fortunata*	344	*Xerocampylaea zelebori*	1159
Pupilla triplicata	727	*Pupilla sterri*	192	*Herilla ziegleri*	551
Cochlodina marisi	463	*Alopia mariae soosi*	52	*Monachoides cf bacescui*	174
Alopia bielzi tenuis	129	*Mastus transsylvanicus*	41	*Campylaea illyrica*	161
Alopia livida julii	17	*Oxychilus montivagus*	16	*Helicopsis instabilis**	290
Mastus bielzi	12	*Alopia grossuana grossuana*	14	*Monacha cartusiana**	17
Carpathica calophana	8	*Pseudalinda viridana*	2	*Vitrea jetschini*	6
Pupilla alpicola	6	*Agardhiella armata*	2	*Orcula jetschini*	1
Orcula dolium	5	*Agardhiella lamellata*	1	*Oxychilus hydatinus*	1
Aegopinella nitens	2	*Helix lucorum**	1	*Soosia diodonta*	1
Succinella oblonga	1				
**East**		**South East**		**Dobrogea**	
Alopia glauca	277	*Alopia lischkeana*	365	*Chondrula microtragus*	1811
Alopia bogatensis bogatensis	137	*Alopia glorifica intercedens*	269	*Strigillaria varnensis*	1587
Alopia glorifica vranceana	129	*Alopia glorifica magnifica*	184	*Oxychilus deilus*	32
Vestia gulo	5	*Alopia plumbea plumbea*	155	*Oxychilus inopinatus*	26
Vestia turgida	5	*Alopia glorifica elegantissima*	137	*Pomatias elegans*	1
Acicula parcelineata	4	*Alopia nefasta ciucasiana*	79		
Pseodotrichia rubiginosa	1	*Alopia nefasta nefasta*	41		
		Oxychilus depressus	29		
		Clausilia pumila	23		
		Lozekia deubeli	10		
		Discus ruderatus	4		
		Columella columella	3		
		Nesovitrea petronella	1		
**ALTITUDE**					
**<300 ex Dobrogea**		**300-500**		**500-700**	
*Helicella instabilis**	290	*Alopia grossuana grossuana*	14	*Alopia vicina fortunatus*	344
Campylaea illyrica	161	*Aegopinella nitens*	2	*Alopia bogatensis bogatensis*	137
*Monacha cartusiana**	17	*Helix lucorum**	1	*Alopia mariae soosi*	52

Vitrea jetschini	6	*Soosia diodonta*	1	*Mastus transylvanicus*	41
Orcula jetschini	1	*Succinella oblonga*	1	*Acicula parcelineata*	4
Oxychilus hydatinus	1			*Pseudalinda viridiana*	2
				Agardhiella lamellata	1
**700-900**		**900+**		**Dobrogea**	
Alopia glorifica magnifica	184	*Alopia lischkeana*	365	*Chondrula microtragus*	1811
Alopia plumbea plumbea	155	*Alopia glorifica intercedens*	269	*Strigillaria varnensis*	1587
Alopia mariae soosi	52	*Zonitoides nitidus*	86	*Oxychilus deilus*	32
Pupilla alpicola	5	*Alopia nefasta ciucasiana*	79	*Oxychilus inopinatus*	26
Vestia turgida	6	*Alopia nefasta nefasta*	41	*Pomatias elegans*	1
Orcula dolium	5	*Clausilia pumila*	23		
Trochulus hispidus	3	*Oxychilus montivagus*	16		
Pseudotrichia rubiginosa	1	*Lozekia deubeli*	10		
		Discus ruderatus	4		
		Columella columella	3		
		Nesovitrea petronella	1		
**WOODY COVER**					
**0-15%**		**20-40%**		**50-60%**	
Alopia glorifica elegantissima	137				
Alopia grossuana	14	*Lozekia deubeli*	10	*Alopia mariae soosi*	52
Vitrea jetschini	6	*Pupilla alpicola*	6	*Succinella oblonga*	1
Discus ruderatus	4	*Orcula dolium*	5		
Soosia diodonta	1	*Columella columella*	3		
Oxychilus hydatinus	1	*Agardhiella lamellata*	1		
*Helix lucorum**	1				
**70-80%**		**85-100%**			
		Alopia plumbea plumbea	155		
Vestia turgida	5	*Alopia nefasta nefasta*	41		
Aegopinella nitens	2	*Pseudalinda viridana*	2		
Orcula jetschini	1	*Agardhiella armata*	2		
		Nesovitrea petronella	1		
		Pseudotrichia rubiginosa	1		
**MICROHABITAT**					
**Open cliff**		**Cliff in Forest**			
Alopia glorifica elegantissima	137	*Alopia mariae soosi*	52		
Alopia grossuana grossuana	14	*Alopia nefasta nefasta*	41		
Vitrea jetschini	6	*Lozekia deubeli*	10		
Nesovitrea hammonis	6	*Columella columella*	3		
Pupilla alpicola	6	*Agardhiella armata*	2		
Orcula dolium	5				
Discus ruderatus	4				
*Helix lucorum**	1				
Soosia diodonta	1				
Oxychilus hydatinus	1				
Agardhiella lamellata	1				
**Large rocks in Forest**		**Small rocks in Forest**			
Alopia plumbea plumbea	155	*Pseudalinda viridiana*	2		
Vestia gulo	5	*Orcula jetschini*	1		
Vestia turgida	5	*Pseudotrichia rubiginosa*	1		
Aegopinella nitens	2				
Nesovitrea petronella	1				
Succinella oblonga	1				

Table S3. The number of species unique to single categories, and subdivided by overall abundance, together with those with occurrence in all categories in each analysis. Dobrogea is excluded.

	Regions	Altitude	Woody %	Microhabitat
Unique total	48	36	20	21
% of Total	36.4	27.3	15.2	15.9
10+ specimens	29	16	4	4
% with 10+	60.4	44.4	20.0	19.0
Universal total	25	32	57	74
% of Total	18.9	24.2	43.2	56.1
